# Synthesis and spectroscopic investigation of a novel sensitive and selective fluorescent chemosensor for Ag^+^ based on a BINOL–glucose derivative†

**DOI:** 10.1039/c8ra04429e

**Published:** 2018-06-26

**Authors:** Yu Hu, Huayin Shen, Xiaohan Zhang, Yang Liu, Xiaoxia Sun

**Affiliations:** Jiangxi Key Laboratory of Organic Chemistry, Jiangxi Science and Technology Normal University Nanchang 330013 China sunxiaoxia77@126.com; College of Chemistry, Nanchang University Nanchang China

## Abstract

Based on a versatile 2,2′-binaphthol (BINOL) backbone, a novel BINOL–glucose derivative fluorescent sensor was synthesized using a click reaction. The fluorescence responses of the BINOL–glucose derivative (*S*,β-d)-1 conclude that it can be used as a specific fluorescent chemical sensor for Ag^+^ in the presence of a large number of competing metal ions without any obvious interference from other metal ions. Mass spectrometric and NMR spectroscopic data were used to study the mechanism, and implied the formation of a 1 + 1 complex between BINOL–glucose 1 and Ag^+^. Both the oxygen atoms of *S*-BINOL and two nitrogen atoms of triazole were involved in coordinating the silver ion.

## Abstract 2

A BINOL–glucose derivative fluorescent sensor was synthesized to detect only Ag^+^ with high selectivity and sensitivity in a 1 + 1 formation.

## Graphical abstract

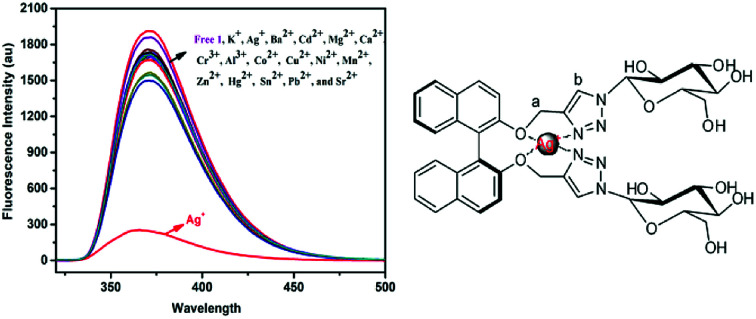

## Introduction

It is very important to discover and design new highly selective fluorescent sensors that can detect heavy metal ions, which may cause serious harm to human health and the living environment. Among the important heavy metals, silver has been paid more attention because of its adverse effects on biological harmony and the environment, due to its ability to easily bind to mercapto groups in proteins and inactivate respiratory enzymes.^1,2^ It is very difficult to identify silver ions from other heavy metal ions because of the moderate coordination ability of Ag^+^. However, almost all fluorescent sensors containing triazole experience interference from other transition metals, especially from mercury ions when attempting to identify silver ions,^3–8^ which leads to the limitation of the use of specific fluorescent sensors for silver ions. Only a few fluorescent sensors^9–13^ that can detect Ag^+^ have been reported in recent years. So, it is still a challenge to design an effective chemical method to target only Ag^+^.

The highly sensitive and rapid identification of isomers and metal ions is the highlight of using optically active BINOL derivatives as a fluorescent chemical sensor. With a versatile BINOL backbone, it can be easily modified through steric stabilization and electronic effects, and due to this, BINOL and its derivatives^14–23^ have attracted great attention in the field of fluorescent chemical sensors^24–30^ and synthetic catalysis. Moreover, sugar is a hot research topic due to its natural existence, good biocompatibility and structural diversity without being toxic. Furthermore, its good water solubility is considered to be a very ideal feature for using it as a component in fluorescent chemical sensors. In recent years, triazole modified functional sugar derivatives have attracted continuous interest in research^31–34^ and various sugar-based fluorescent chemosensors have been synthesized using click reactions. In this way, a novel BINOL–glucose derivative fluorescent sensor was synthesized through the Cu-catalyzed 1,3-dipolar cycloaddition^35–40^ of an alkyne and azole. The synthesis was based on a versatile (*S*)-BINOL backbone, and the (*S*)-BINOL and two triazole units were used to represent the fluorophore and recognition group, respectively. The synthesized sensor could then be used as a specific fluorescent chemical sensor for Ag^+^ in the presence of a large number of competing cations, as anticipated.

## Experimental

### Materials and methods

All the solvents were of analytical purity, and the materials were obtained from commercial suppliers or prepared by our laboratory, with no further purification of the commercially suppled chemicals before use. If not otherwise specified, the solvents used in the optical spectroscopic studies were of spectroscopic purity. Various metal ion solutions (0.1 M) were prepared from their respective nitrates in distilled-deionized water, except for K^+^, Hg^2+^, Mn^2+^, and Ba^2+^, which were made from their chloride salts. AgNO_3_ was used as the Ag^+^ source unless otherwise stated. ^1^H nuclear magnetic resonance (NMR) and ^13^C NMR were measured on a Bruker AM-400WB spectrometer using tetramethylsilane (TMS) as an internal standard and CDCl_3_ or CD_3_OD as solvents. All UV-Vis absorptions were recorded on an Agilent 8453 UV-Vis spectrometer. Fluorescence emission spectra were obtained using a Hitachi F-4500 fluorescence spectrometer at 298 K, unless otherwise stated. Electrospray ionization mass spectrometric (ESI-MS) data were recorded using a Thermo Fisher LCQ. Melting points were measured on a WRS-1B melting point apparatus. Optical rotation was carried out using a Rudolph AUTOPOL IV automatic polarimeter.

### Synthesis of (*S*,β-d)-2

*S*-2,2′-Bis(prop-2-yn-1-yloxy)-1,1′-binaphthalene (0.63 g, 1.75 mmol) and 2,3,4,6-tetra-*O*-acetyl-beta-d-glucopyranosyl azide (1.4 g, 3.67 mmol) were added to 50 mL of tetrahydrofuran (THF) with stirring at 273 K under an argon atmosphere and the mixture was stirred for five minutes. Sodium ascorbate (0.69 g, 3.48 mmol) and CuSO_4_·5H_2_O (0.44 g, 1.76 mmol) were added to the mixture and the temperature slowly rose to room temperature, after which it was stirred for 12 h under Ar_2_. After the reaction was completed, the mixture was poured into ice water. The mixture was extracted three times with EtOAc and then the organic layer was washed with brine and dried over anhydrous MgSO_4_. After evaporation of the organic solvent, the crude product was purified directly by column chromatography on silica (petroleum ether : EtOAc = 1 : 1, v/v) to give the desired product (*S*,β-d)-2 (1.7 g, 41.9%) as a white solid: [*α*]^25^_D_ – 41.2 (*c* 0.1, CH_3_OH). ^1^H NMR (400 MHz, CDCl_3_) *δ* 7.99 (d, *J* = 9.0 Hz, 1H), 7.93 (d, *J* = 8.1 Hz, 1H), 7.49 (d, *J* = 9.0 Hz, 1H), 7.39 (t, *J* = 7.4 Hz, 1H), 7.25 (d, *J* = 7.0 Hz, 1H), 7.19–7.12 (m, 2H), 5.79 (d, *J* = 9.4 Hz, 1H), 5.42–5.30 (m, 2H), 5.25 (t, *J* = 9.2 Hz, 1H), 5.19 (s, 2H), 4.30 (dd, *J* = 12.6, 5.1 Hz, 1H), 4.13 (d, *J* = 12.7 Hz, 1H), 3.99 (dd, *J* = 9.5, 3.1 Hz, 1H), 2.11–2.02 (m, 9H), 1.73 (d, *J* = 20.0 Hz, 4H). ^13^C NMR (400 MHz, CDCl_3_) *δ* 170.32, 169.57, 168.55, 153.68, 145.53, 133.93, 131.53–130.59, 130.04, 128.87, 127.29, 126.91–124.70, 124.70–124.5, 123.23, 122.24, 120.43, 116.66, 115.07, 86.28, 84.69, 77.42, 77.10, 76.78, 75.63, 74.22, 73.88, 72.06, 71.00, 69.46, 68.38, 66.85, 65.10, 63.41, 61.94, 60.21, 22.60, 21.95, 21.30, 20.3, 19.97–19.70, 19.70–17.92 ppm. HRMS (ESI^−^): calcd for [C_54_H_56_N_6_O_20_ + Cl]^−^ 1143.8; found 1143.3.

### Synthesis of (*S*,β-d)-1

A mixture of (*S*,β-d)-2 (1.3 g, 1.17 mmol), NaOH (0.47 g, 11.7 mmol), and methanol (100 mL) was stirred at room temperature for 12 h. After the reaction was completed, the solvent was removed under reduced pressure to obtain the crude product. The product was purified directly by flash column chromatography on silica gel using dichloromethane and methanol in a ratio of 2 : 1 (v/v) as the eluent to give the desired product 1 (0.75 g, 82.8%) as a white solid:[*α*]^25^_D_ – 54 (*c* 0.07, CH_3_OH). ^1^H NMR (400 MHz, MeOD) *δ* 8.02 (d, *J* = 6.3 Hz, 1H), 7.92 (d, *J* = 8.1 Hz, 1H), 7.69–7.54 (m, 1H), 7.34 (d, *J* = 9.8 Hz, 2H), 7.23 (s, 1H), 7.06 (d, *J* = 8.5 Hz, 1H), 5.45 (d, *J* = 9.1 Hz, 1H), 5.27–5.04 (m, 2H), 3.87 (d, *J* = 10.9 Hz, 1H), 3.68 (dd, *J* = 10.8, 6.9 Hz, 2H), 3.62–3.41 (m, 3H), 3.31 (s, 4H) ppm. ^13^C NMR (101 MHz, MeOD) *δ* 153.77, 144.31, 133.93, 129.77, 129.49, 127.90, 125.73, 125.16, 123.86, 122.80, 120.48, 115.77, 87.99, 79.65, 77.19, 72.65, 69.82, 63.15–62.26, 61.12 ppm. HRMS (ESI^−^): calcd for [C_40_H_44_N_6_O_12_ + Cl]^−^ 807.2; found 807.2.

### Fluorescence and UV-visible measurements

A stock solution of (*S*,β-d)-1 (2.0 × 10^−5^ mol L^−1^) prepared in methanol and stock solutions of the metal ions (0.1 mol L^−1^ M in H_2_O) were freshly prepared before testing each performance. For each fluorescence quenching measurement, varying equivalents of Ag^+^ stock solution were added to the sensor solutions in a 5 mL volumetric flask at 298 K. The competition experiments involved mixing of each metal ion solution with a stock solution of Ag^+^ of the same concentration (2.0 × 10^−5^ mol L^−1^). The Job plot for the complexation of (*S*,β-d)-1 with Ag^+^ was obtained by recording the fluorescence response of (*S*,β-d)-1 with different ratios of Ag^+^. A 2.0 mM stock solution of (*S*,β-d)-1 dissolved in methanol and a 2.0 mM AgNO_3_ in H_2_O were freshly prepared for each measurement.

### Preparation of the samples for NMR tests

AgNO_3_ (0.1 M in D_2_O) was gradually added to an NMR tube containing (*S*,β-d)-1 (0.4 mL, 25 mM) to obtain 1 : 0, 1 : 0.1, 1 : 0.3, 1 : 0.5, 1 : 0.7, and 1 : 1 ratios of the sensor to Ag^+^. The resulting solutions were allowed to equilibrate at 298 K for 4 h before testing.

## Results and discussion

**Scheme 1:**
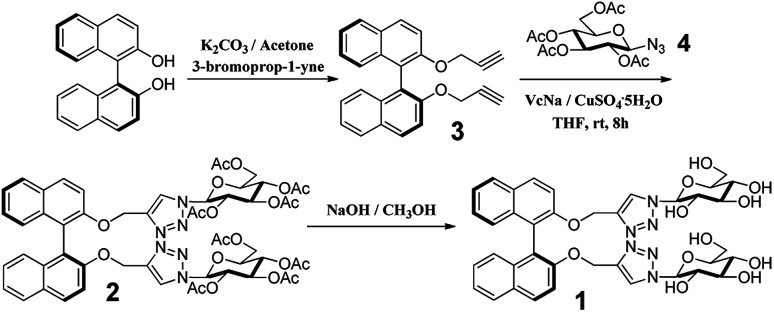
Synthetic route for compound (*S*,β-d)-1.

The BINOL–glucose derivative fluorescent sensor was synthesized using “click chemistry”, as shown in Scheme 1. According to the previous literature, the dipropargyl 3 (ref. 41) derivative of (*S*)-BINOL was prepared (Scheme 1). The reaction of dipropargyl 3 and azide-functionalized glucose 4 was carried out in THF at room temperature in the presence of sodium ascorbate and copper(ii) sulfate to afford BINOL–glucose derivative 2 in moderate yield after a simple purification process. The new fluorescent sensor 1 was obtained when 2 was hydrolyzed in methanol, in 83% yield. The structures of the desired compounds were determined from ^1^H NMR, ^13^C NMR, and ESI-MS measurements.

### Fluorescence study

**Fig. 1:**
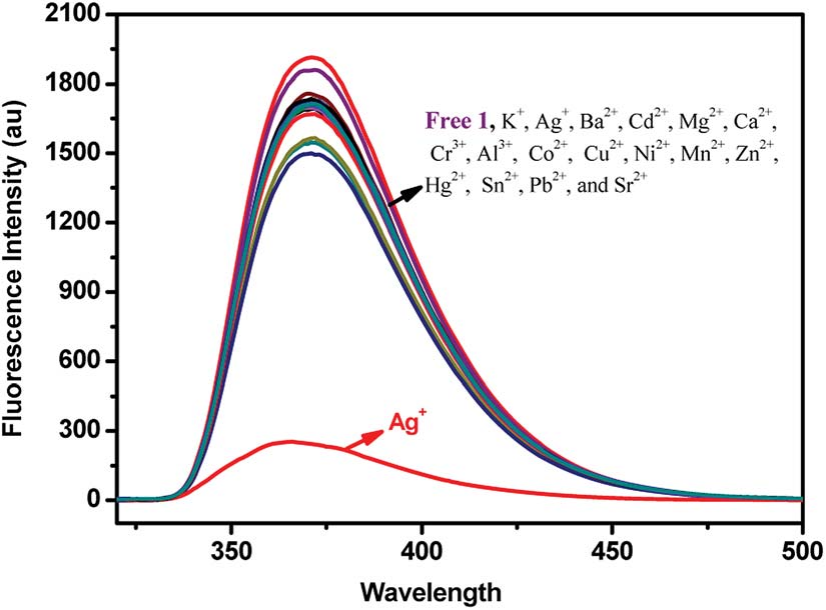
Fluorescence spectra of 1 (2 × 10^−5^ mol L^−1^ in CH_3_OH) upon the addition of K^+^, Ag^+^, Ba^2+^, Cd^2+^, Mg^2+^, Ca^2+^, Cr^3+^, Al^3+^, Co^2+^, Cu^2+^, Ni^2+^, Mn^2+^, Zn^2+^, Hg^2+^, Mn^2+^, Sn^2+^, Pb^2+^, and Sr^2+^ ions (5 equiv.).

The fluorescence responses of (*S*,β-d)-1 were studied in the presence of Ag^+^, Ba^2+^, Cd^2+^, Mg^2+^, Ca^2+^, Cr^3+^, Al^3+^, Ca^2+^, Co^2+^, Cu^2+^, K^+^, Ni^2+^, Mn^2+^, Zn^2+^, Hg^2+^, Sn^2+^, Pb^2+^, and Sr^2+^ ions in different solutions using fluorescence spectroscopy. First, the fluorescence measurements of (*S*,β-d)-1 were carried out in CH_3_OH ([(*S*,β-d)-1] = 20 μM). As shown in Fig. 1, only the addition of Ag^+^ ions to a solution of (*S*,β-d)-1 could result in an almost complete quenching of the fluorescence, however, other metal ions induced no obvious change in the fluorescence response. When the fluorescence selectivity experiments of (*S*,β-d)-1 were tested in THF solution (as shown in Fig. S8†), no significant difference in the fluorescence response was found. The discrimination between the different metal ions showed a sensitive dependence on the solvent. So, all of the fluorescence measurements of (*S*,β-d)-1 were investigated in methanol solution, in which (*S*,β-d)-1 was found to be highly selective and sensitive towards Ag^+^. An unexpected quenching of the fluorescence of the excimer emission upon addition of Ag^+^ ions to a solution of 1 may be due to PET (photoinduced electron transfer). In other words, the metal ions combined with electron acceptor triazole units and the glucose units behaved as a PET donor. This means that the two triazole units of (*S*,β-d)-1 form a selective and effective metal ion binding site.

### Metal ion competition studies

**Fig. 2:**
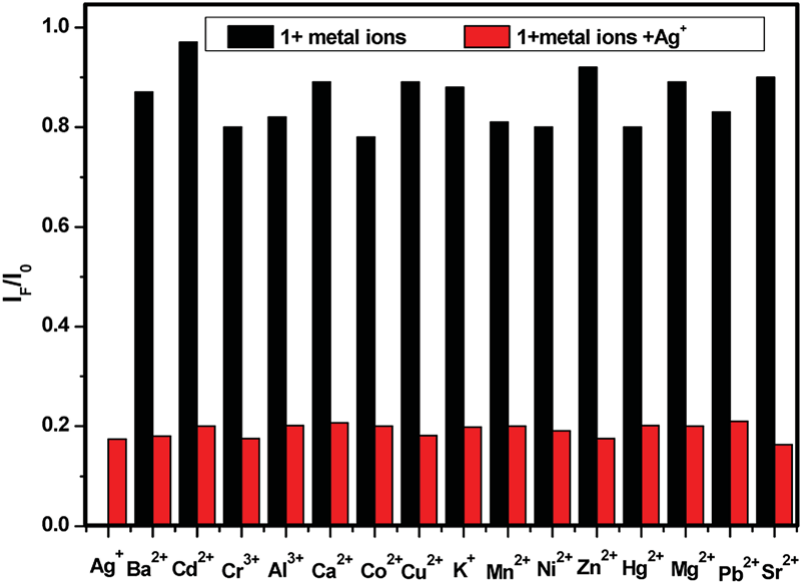
Fluorescence quenching degrees in terms of the *I*_F_/*I*_0_ ratio of 1 (2 × 10^−5^ mol L^−1^ in methanol) in the presence of both Ag^+^ (5.0 equiv.) and competing metal ions (5.0 equiv.). The black bars represent the addition of 5 equiv. of various metal ions to a solution of 1, and the red bars represent the competing metal ion added in the presence of Ag^+^.

For an effective cation probe, the key factor is the ability to detect a specific metal ion in the presence of different metal ions. Fig. 2 shows the results of competition experiments we conducted in which we tested the ability of the probe to selectively detect one metal ion over other metal ions. The addition of 5.0 equiv. of Ag^+^ combined with 5.0 equiv. of the other metal ions in methanol solutions of (*S*,β-d)-1, respectively, were used in the competitive experiments. The quenching ratio of the *I*_F_/*I*_0_ value at 375 nm (where *I*_0_ represents the fluorescence intensity of only (*S*,β-d)-1 and *I*_F_ represents the fluorescence intensity upon the addition of a mixture of competitive metal ions and Ag^+^) for the majority of the competitive metal ions was almost 0.17. No significant interference was observed in the presence of the various competitive metal ions. This indicated that the BINOL–glucose sensor could be used to detect Ag^+^ in the presence of various competitive ions without any obvious interference from the other metal ions. Based on the fluorescence results, we found that the BINOL–glucose derivative has high selectivity and sensitivity to Ag^+^ and therefore can be used as an Ag^+^ sensor.

**Fig. 3:**
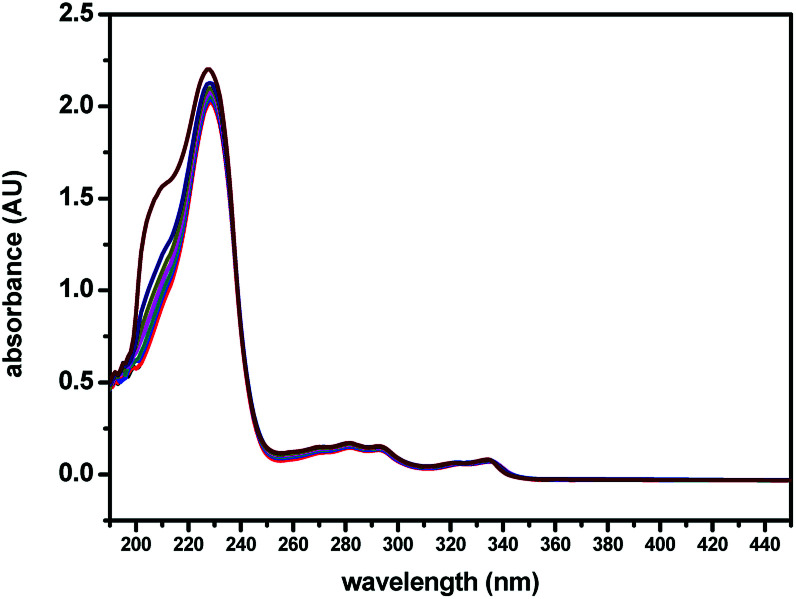
UV-Vis spectroscopic titrations of (*S*,β-d)-1 (20 μM) with various equivalents of Ag^+^ (0, 0.25, 0.5, 0.75, 1, 1.5, 2, 3 equiv.) in CH_3_OH.

The binding tests of (*S*,β-d)-1 towards Ag^+^ were also studied upon the addition of different amounts of Ag^+^ to (*S*,β-d)-1 in CH_3_OH, using UV-Vis absorption spectroscopy. As shown in Fig. 3, the maximum absorption wavelength of (*S*,β-d)-1 was found to be around 229 nm upon the addition of 0 to 3 equivalent of Ag^+^. However, there was found to be a slight change in the UV-Vis absorption spectrum of (*S*,β-d)-1 in the presence of various concentrations of Ag^+^, where the absorbance at 210 nm was observed to begin to increase, which may indicate the formation of a (*S*,β-d)-1–Ag^+^ complex. It is a pity that the slight absorbance intensity shift did not induce any obvious color change. The interaction between the host and guest was evaluated using fluorescence spectroscopy. From Fig. 3, it can be seen that (*S*,β-d)-1 has three absorption peaks at 229 nm, 290 nm and 335 nm. The maximum absorption wavelength *λ*_max_ of (*S*,β-d)-1 was around 229 nm with weak fluorescence emission. However, the absorption at 290 nm was observed to have a stronger fluorescence intensity (see Fig. S9†), and so, *λ*_ex_ = 290 nm.

**Fig. 4:**
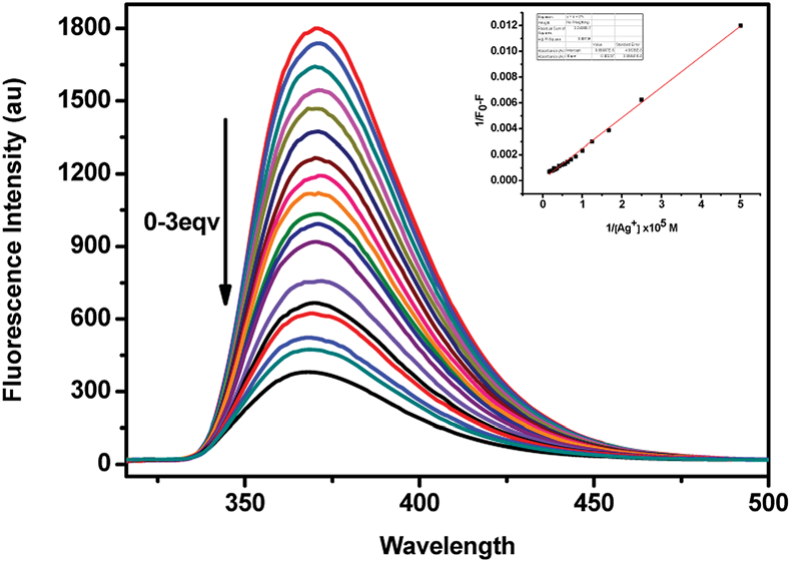
Fluorescence spectra of 1 (2 × 10^−5^ mol L^−1^ in CH_3_OH, *λ*_ex_ = 290 nm) in the presence of an increasing amount of 0–3 equiv. of Ag^+^ (0.01 M). The inset shows the plot of 1/(*F*_0_ − *F*) *versus* 1/[Ag^+^].

**Fig. 5:**
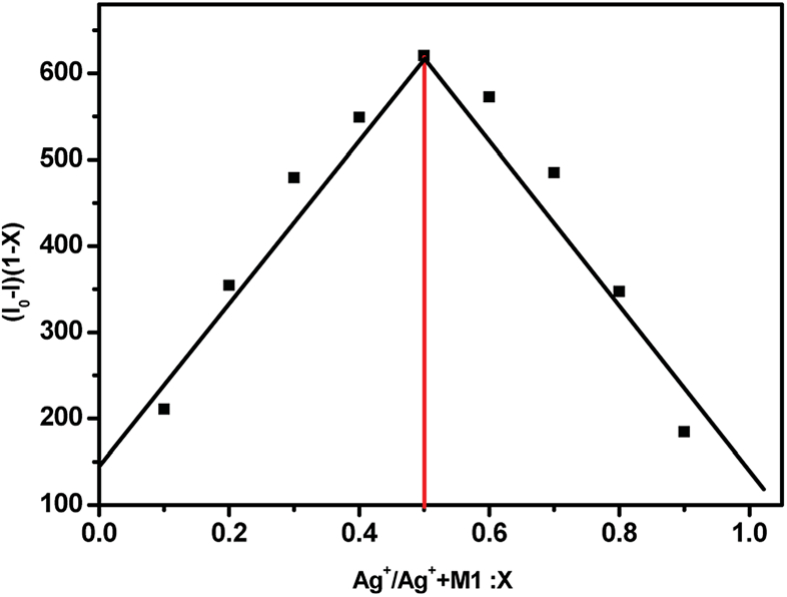
The Job plot of a 1 : 1 complex of (*S*,β-d)-1 with Ag^+^ on the basis of the fluorescence signal. *X* represents the molar fraction of Ag^+^. The total concentration of (*S*,β-d)-1 and Ag^+^ was found to be 2.0 × 10^−5^ M.

As shown in Fig. 4, the fluorescence spectra of (*S*,β-d)-1 upon adding different equivalents of Ag^+^ were used to determine the nature of the complex formed between (*S*,β-d)-1 and Ag^+^. When the amount of Ag^+^ used was above 3 equiv., the intensity of the fluorescence emission underwent a strong change. We assumed that the stoichiometry ratio of (*S*,β-d)-1–Ag^+^ complex is 1 : 1, so the association constant *K* of (*S*,β-d)-1 with Ag^+^ was found to be 2.4 × 10^6^ M^−1^ (*R* = 0.997) from the Lineweaver–Burk plot of 1/(*F*_0_ − *F*) *versus* 1/[Ag^+^]. As shown in Fig. 5, the maximum fluorescence quenching of (*S*,β-d)-1 provided by Ag^+^ takes place at a ratio of 1 : 1. Based on the fluorescence analyses, the BINOL–glucose compound and Ag^+^ were proposed to form a 1 + 1 complex. The Job plot for the complex provided further direct evidence that the (*S*,β-d)-1–Ag^+^ complex stoichiometry ratio was 1 : 1. Respectively, the detection limit (LOD) of (*S*,β-d)-1 to Ag^+^ was assessed to be 1.57 × 10^−9^ mol L^−1^ using the following equation: LOD = 3*σ*/*s*, where *σ* represents the standard deviation of the (*S*,β-d)-1 solution and *s* represents the slope between the fluorescence intensity *versus* the Ag^+^ concentration (Fig. S11†). Further evidence for the 1 : 1 complex stoichiometry ratio was obtained from ESI-MS spectra data (see Fig. S7, ESI data†). The peak at *m*/*z* = 881.1 (calcd 881) was found to correspond to [1–Ag^+^ + H^+^], which supports our assumption there is strong binding between (*S*,β-d)-1 and Ag^+^.

**Fig. 6:**
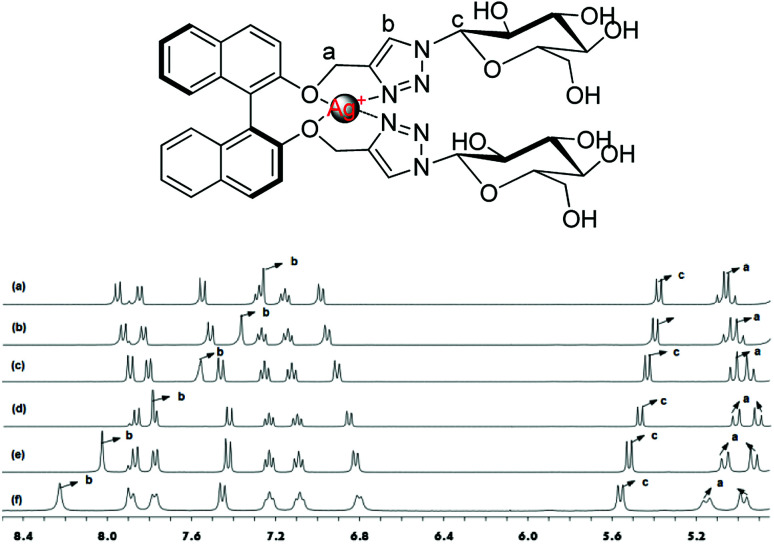
^1^H NMR spectra of (a) 1; (b) the addition of 0.1 equiv. of Ag^+^; (c) the addition of 0.3 equiv. of Ag^+^; (d) the addition of 0.5 equiv. of Ag^+^; (e) the addition of 0.7 equiv. of Ag^+^; (f) the addition of 1 equiv. of Ag^+^.

The ^1^H NMR experiments were carried out in CD_3_OD to find out further detailed information on the binding of Ag^+^ with (*S*,β-d)-1. As shown in Fig. 6, different equiv. (from 0 to 1 equiv.) of Ag^+^ were added to a solution of (*S*,β-d)-1 and resulted in an obvious downshift in the chemical shifts, however, the shifts tended to saturation when the amount of Ag^+^ was beyond 1 equiv. The H_a_ nuclear magnetic peaks of the –OCH_2_– linking triazole groups are split into two sets of signals; a group of displacement peaks migrate Δ*δ* 0.11 ppm downfield from 5.05 ppm to 5.16 ppm, while another group of displacement peaks shift up field by 0.09 ppm, from 5.05 ppm to 4.96 ppm. These changes demonstrate that Ag^+^ is selectively bound to the BINOL–glucose sensor through the oxygen atoms on the BINOL. The H_c_ peak of the glucose linked to the triazole groups exhibited a smaller chemical shift from 5.31 ppm to 5.54 ppm. In particular, the H_b_ peak of the triazole rings was observed to undergo an obvious downshift of Δ*δ* = 0.97 ppm from 7.26 ppm to 8.23 ppm. From the above data, we concluded that both of the oxygen atoms of the BINOL and the nitrogen atoms on the triazole of (*S*,β-d)-1 were involved in the formation of a tetrahedral complex with a silver ion at the center. The results obtained from fluorescence and NMR spectroscopic and mass spectrometric analyses were also used to confirm the recognition between the BINOL–glucose compound and Ag^+^ in a 1 + 1 complex.

## Conclusions

In summary, through a four step reaction, a novel highly selective and sensitive BINOL–glucose derivative fluorescent sensor (*S*,β-d)-1 that can detect Ag^+^ was synthesized with an overall yield of 30%. (*S*,β-d)-1 was observed to function as an Ag^+^ specific fluorescent sensor, as it showed high sensitivity and specificity in the experiments. The highly selective fluorescence turn-off behavior was caused by the formation of a 1 + 1 binding complex between BINOL–glucose 1 and Ag^+^ without any interference from various different metal ions. The fluorescence quenching was ascribed to both the triazole nitrogen atoms and the oxygen atoms of (*S*,β-d)-1 involved in the coordination with Ag^+^. These results show the possibility of preparing highly selective fluorescent sensors based on the versatile BINOL backbone for the detection of various metal ions.

## Conflicts of interest

There are no conflicts to declare.

## Supplementary Material

RA-008-C8RA04429E-s001
