# Supplementary material for: Synthesis and spectroscopic investigation of a novel sensitive and selective fluorescent chemosensor for Ag+ based on a BINOL–glucose derivative
Source: RSC Adv. 2018 Jun 26;8(41):23252–6. doi: 10.1039/c8ra04429e (PMC9081579; doi:10.1039/c8ra04429e)
Supplement: RA-008-C8RA04429E-s001 [file RA-008-C8RA04429E-s001.pdf]

Electronic Supplementary Material (ESI) for RSC Advances.
This journal is © The Royal Society of Chemistry 2018

Supporting Information

A Novel Sensitive and Selective Fluorescent Chemosensor for Ag^+ based on BINOL-Glucose Derivative and Spectroscopic Investigation

Yu Hu, [a b] Huayin Shen,[a] Xiaohan Zhang,[a] Yang Liu,[a] and Xiaoxia Sun*[a]

[a] Jiangxi Key Laboratory of Organic Chemistry, Jiangxi Science and Technology Normal University, Nanchang, 330013, China

[b] College of Chemistry, Nanchang University, Nanchang, China
Email: sunxiaoxia77@126.com

Contents

1 Synthesis of 2,3,4,6-Tetra-O-acetyl-alpha-D-glucopyranosyl bromide……………………S2
2 Synthesis of 2,3,4,6-Tetra-O-acetyl-beta-D-glucopyranosyl azide………………………S2
3 ^{1}HNMR, ^{13}CNMR of **(*S*, *β*-*D*)-2**($CDCl_3$)..S3
4 ^{1}HNMR, ^{13}CNMR of **(*S*, *β*-*D*)-1**(CD_3OD)...S4
5 ESI-MS of **(*S*, *β*-*D*)-2 and (*S*, *β*-*D*)-1**...S5
6 ESI-MS of **[(*S*, *β*-*D*)-1+Ag^++H^+]**...S6
7 Fluorescence spectra of (*S*, *β*-*D*)-**1** (2 × 10^{-5} mol/L in THF) S6
8 UV-vis spectra titration and Fluorescence spectra Fluorescence spectra of **(*S*, *β*-*D*)-1**........S7
9 Hildebrand-Benesi plot base on (*S*, *β*-*D*)-1..S8
10 The limit of detection (LOD) S8

Synthesis of 2,3,4,6-Tetra-O-acetyl-alpha-D-glucopyranosyl bromide

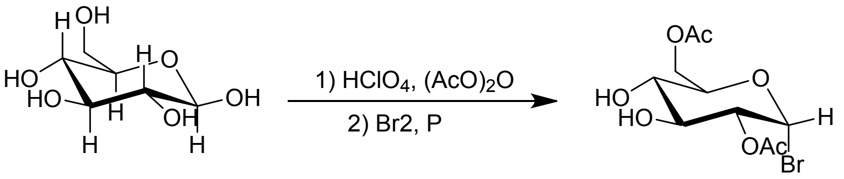

At 273 K, perchloric acid (0.2 mL, 0.2 mol) was added slowly to acetic anhydride (25 mL, 0.25 mol) in 100 mL flask. After stirring for 5 min, glucose (5 g) and 0.03 phosphorus (5 g, 0.45 mol) was separated added to the flask and the mixture was stirred for another 10 min, then Br2 (3 mL, 0.12 mol) was added slowly. The mixture was slowly rises to the room temperature reaction of 1 h. At 273K, 3 mL of water was added to the reaction system and the reaction was stirred 2 h. Finally, 20 mL CH_2Cl_2 was added and the mixture was then poured into 100 mL ice water. Filter out excess red phosphorus, separate the organic phase, clean the water layer with CH_2Cl_2 for three times, combine organic phase, and wash it to neutral with saturated Na_2CO_3 solution. Wash with saturated salt water and dry MgSO4 without water. Filter and spin. Finally, the white solid 9.8 g was obtained by recrystallization with ether petroleum ether, and the yield was 86%.^{1}HNMR(400 MHz, $CDCl_3$) δ 6.61 (d, J = 9.5 Hz, 1H), 5.56 (d, J = 9.8 Hz, 1H), 5.14 (s, 1H), 4.83 (d, J = 8.9 Hz, 1H), 4.31 (s, 1H), 4.12 (s, 1H), 4.08 (s, 1H), 2.06 (dd, J = 28.0, 9.5 Hz, 12H).

Synthesis of 2,3,4,6-Tetra-O-acetyl-beta-D-glucopyranosyl azide

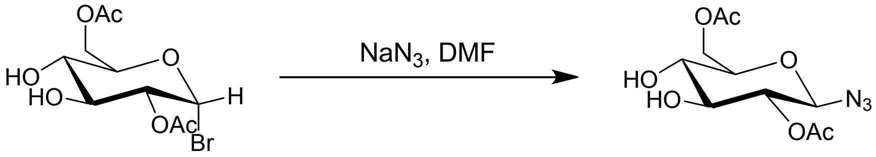

NaN_3(3.2 g, 49.85 mmol）was added to 2,3,4,6-Tetra-O-acetyl-alpha-D-glucopyranosyl bromide in 50 ml DMF under Ar_2. The mixture was stirred for 12 h at room temperature and then pured into 200 ml ice-water. White solid was appeared quickly under hardly stirred. Filtered and washed with cold water. White solid 2.7 g was obtained with 73% yield. ^{1}HNMR (400 MHz, $CDCl_3$) δ 5.21 (d, J = 9.5 Hz, 1H), 5.12 (d, J = 9.8 Hz, 1H), 4.96 (s, 1H), 4.65 (d, J = 8.9 Hz, 1H), 4.27 (s, 1H), 4.19 (s, 1H), 3.81 (s, 1H), 2.06 (dd, J = 28.0, 9.5 Hz, 12H).

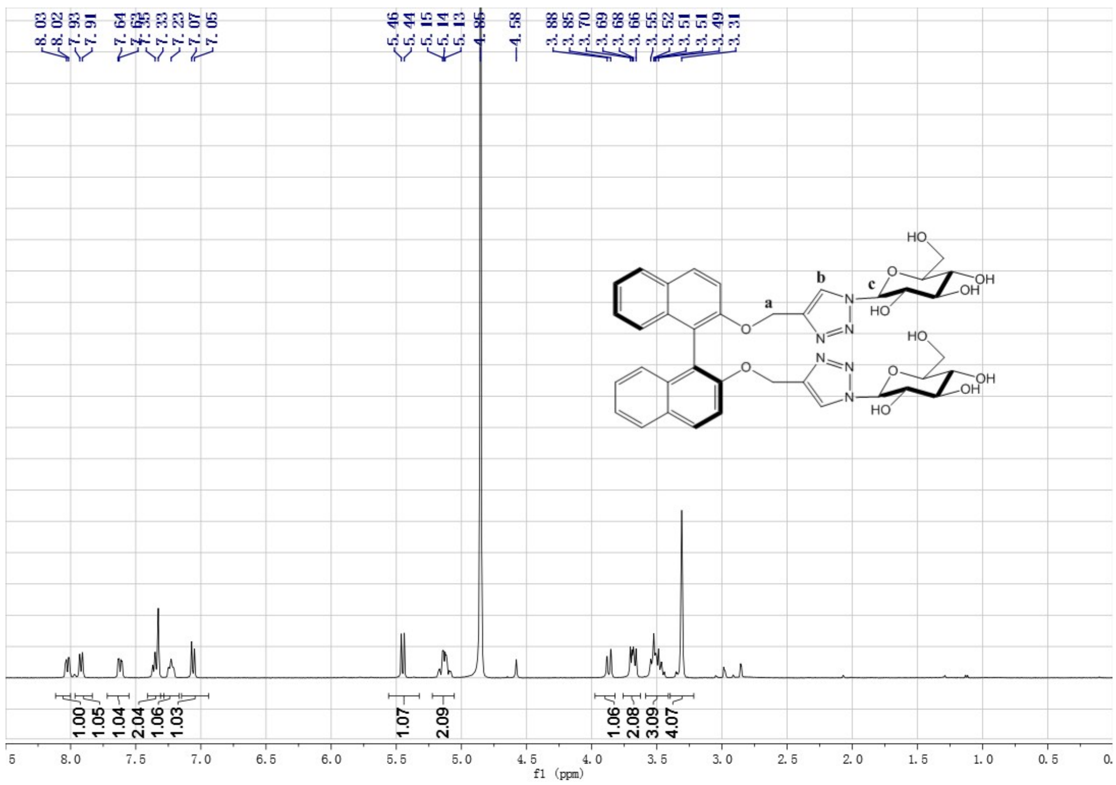

Figure S1 ^{1}HNMR of ***(S, β-D)*-1**(CD_3OD)

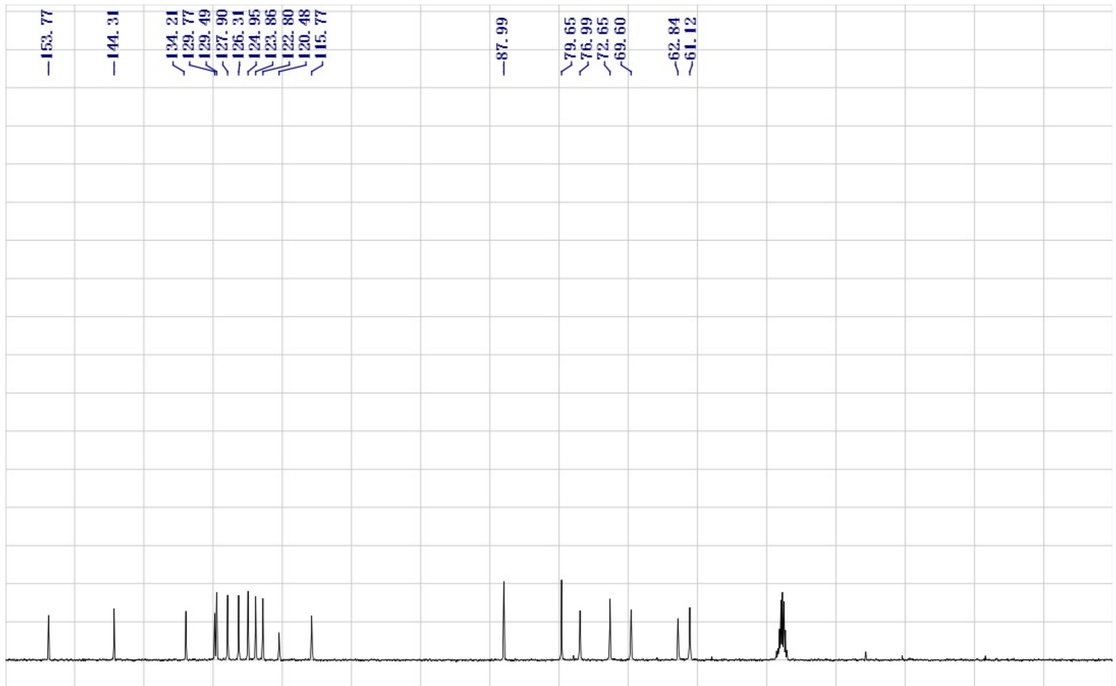

Figure S2 ^{1}HNMR, ^{13}CNMR of ***(S, β-D)*-1**(CD_3OD)

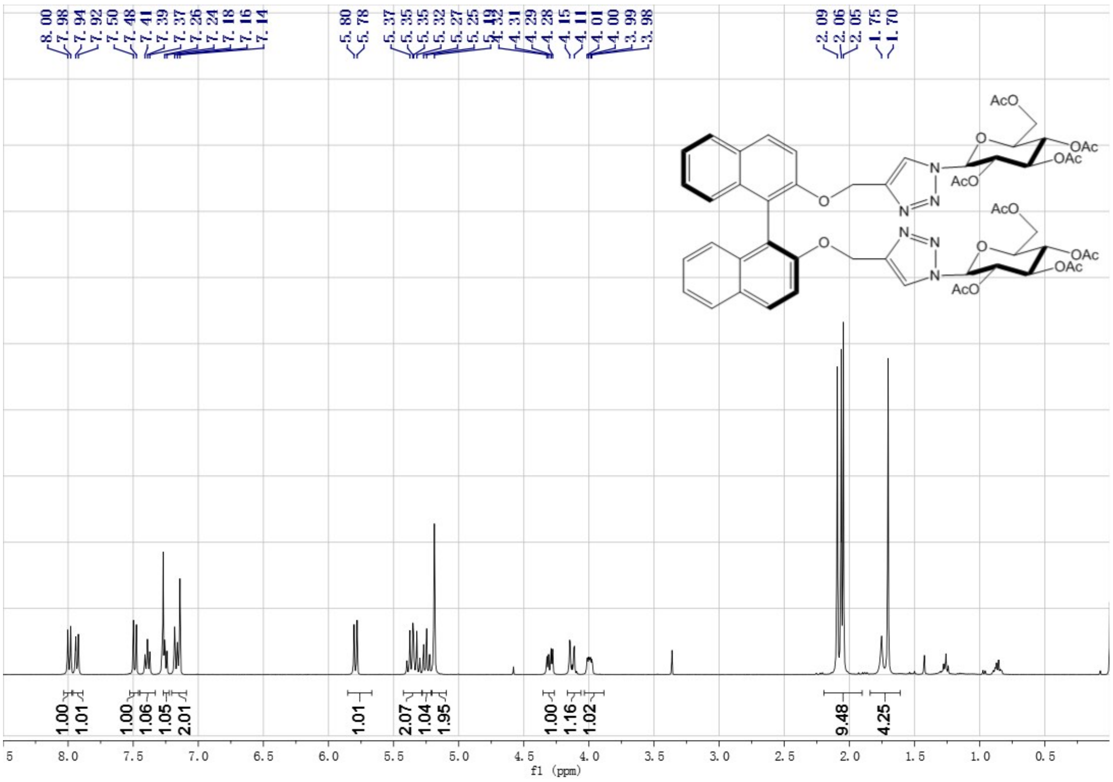

Figure S3 ^{1}HNMR of ***(S, β-D)*-2**($CDCl_3$)

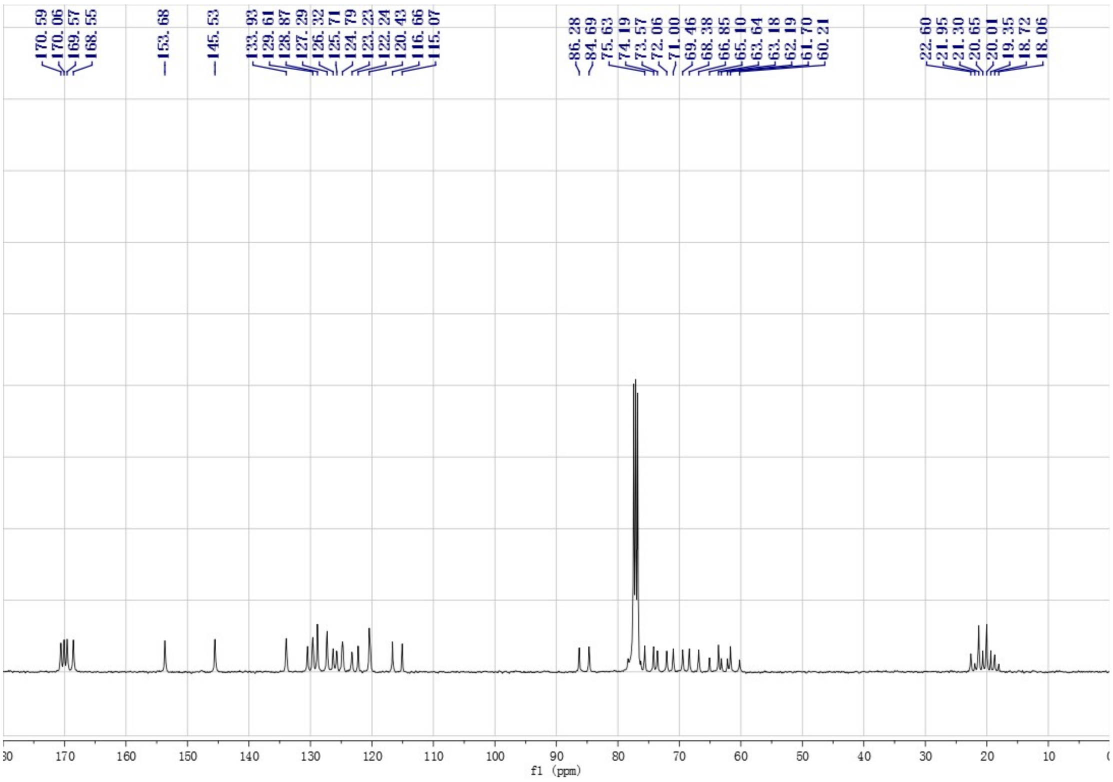

Figure S4 ^{13}CNMR of **(*S*, *β-D*)-2**($CDCl_3$)

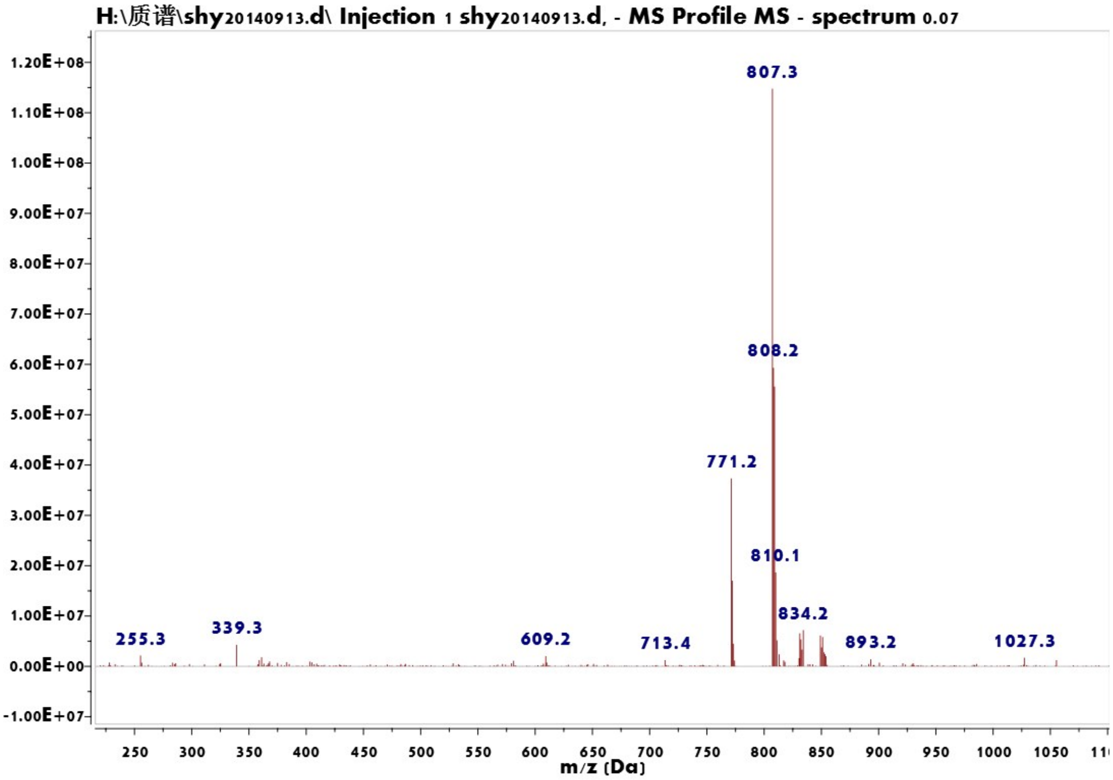

Figure S5 ESI-MS of **(*S*, *β-D*)-1**

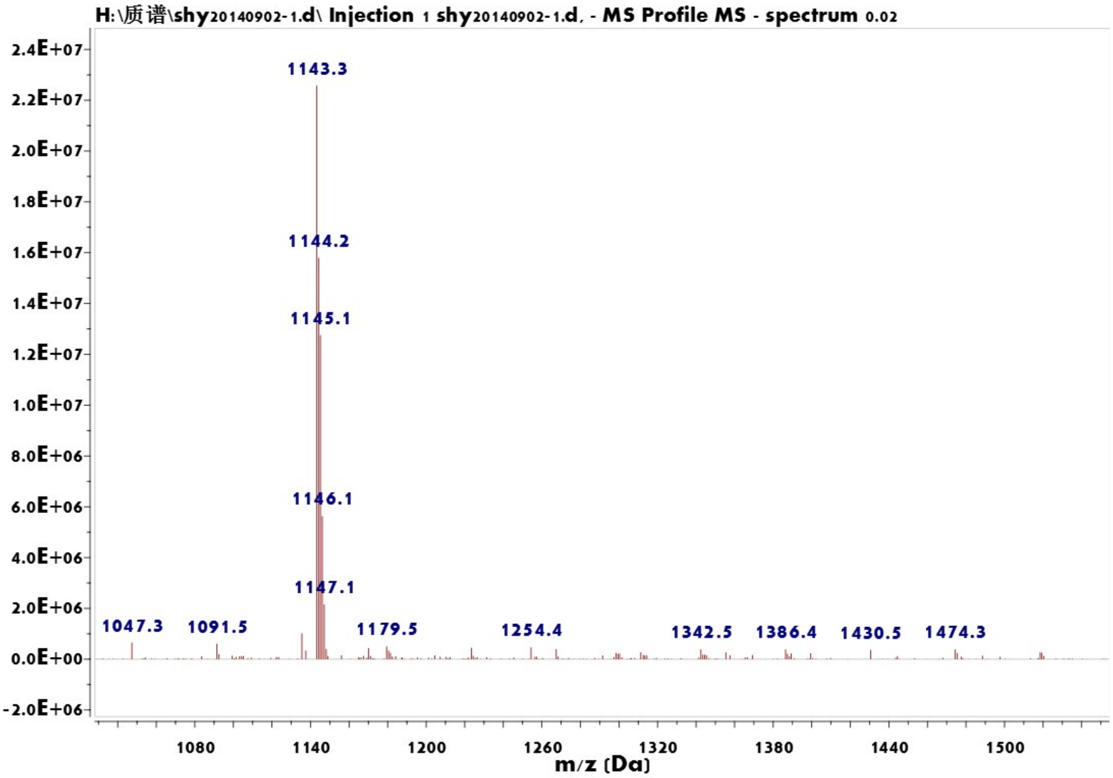

Figure S6 ESI-MS of **(*S*, *β-D*)-2**

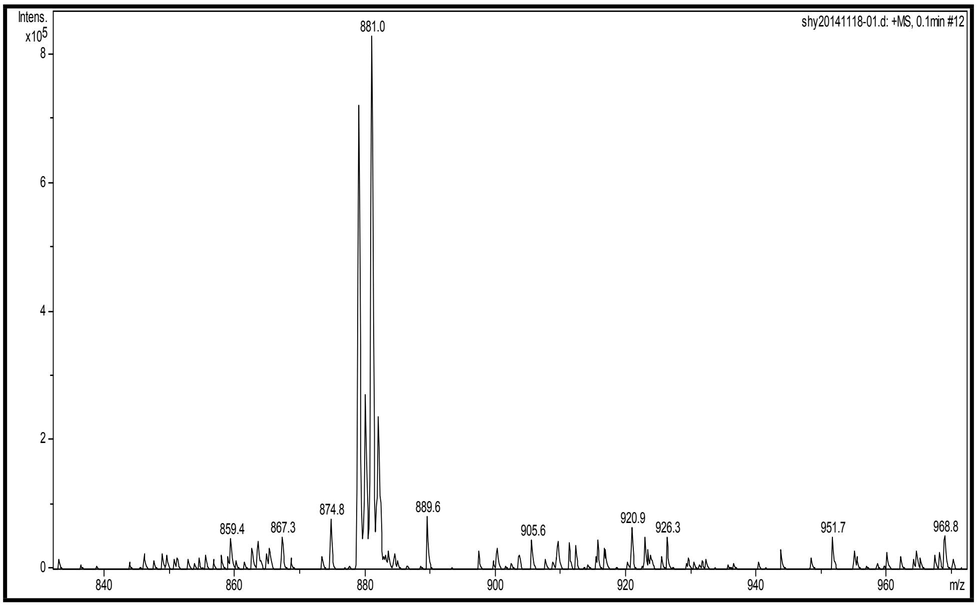

Figure S7. ESI-MS of [1-Ag^{+}+H^{+}]

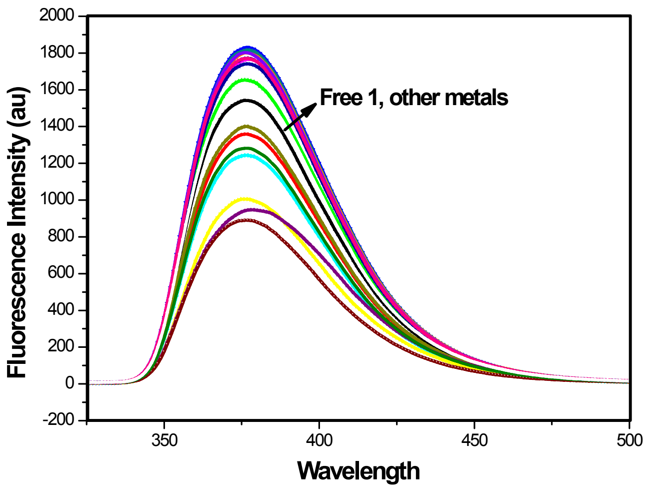

Figure S8 Fluorescence spectra of (*S, β-D*)-**1** (2 × 10^{-5} mol/L in THF) in presence of various ions such as K^{+}, Ag^{+}, Ba^{2+}, Cd^{2+}, Mg^{2+}, Ca^{2+}, Cr^{3+}, Ni^{2+}, Mn^{2+}, Cu^{2+}, Zn^{2+}, Co^{2+}, Hg^{2+}, Al^{3+}, Sn^{2+}, Pb^{2+}, and Sr^{2+} ions (5 equiv).

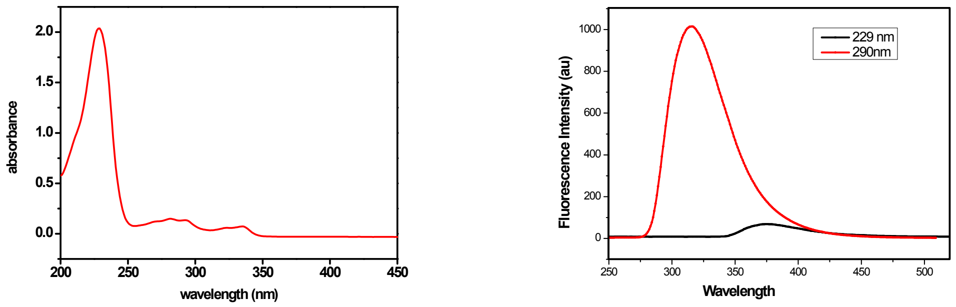

Figure S9 (a) UV-vis spectra titration of (*S, β-D*)-1 (2×10^{-5} mol/L in CH_3OH)
(b) Fluorescence spectra of (*S, β-D*)-1 (2×10^{-5} mol/L in CH_3OH)

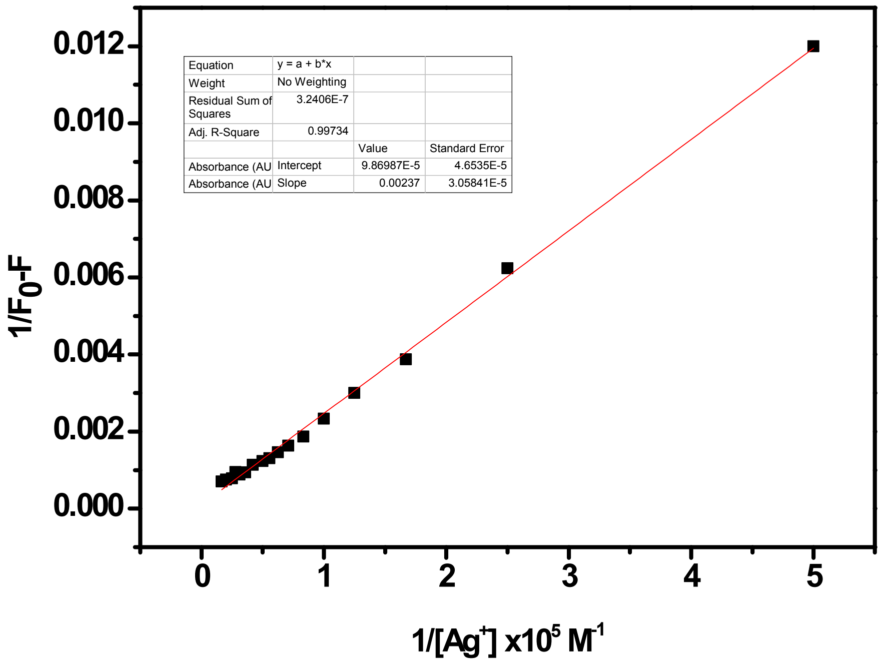

Figure S10 Hildebrand-Benesi plot base on (*S, β-D*)-1 (2×10^{-5} mol/L in CH_3OH, λ_{ex} =290 nm) in the presence of increasing amount 0-3 equiv Ag^+ (0.01M), the binding constant is 2.4×10^6 M^{-1}.

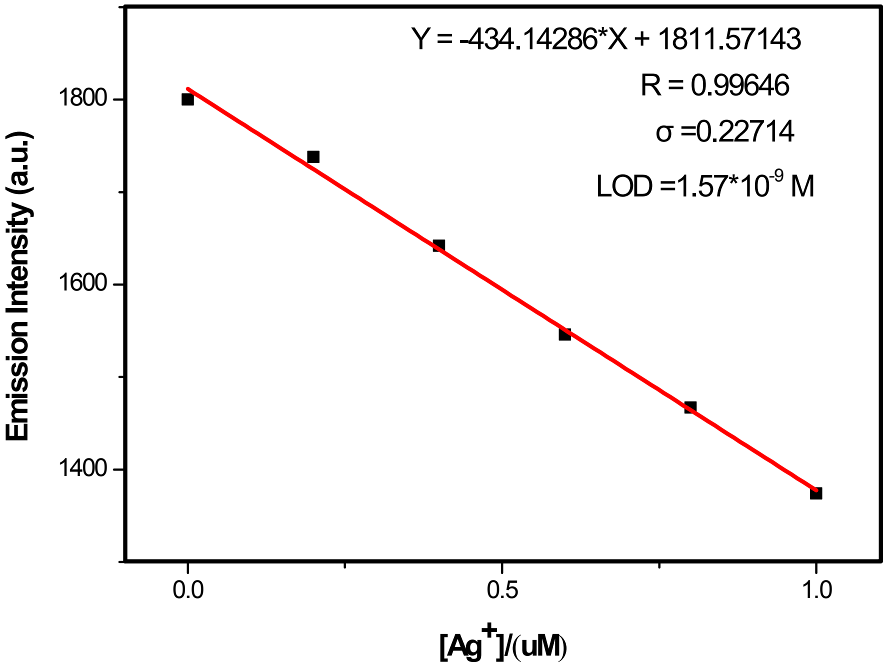

Figure S11 The limit of detection (LOD), LOD is 1.57×10^{-9} M.
